# Antibody-mediated immunity to the obligate intracellular bacterial pathogen *Coxiella burnetii *is Fc receptor- and complement-independent

**DOI:** 10.1186/1471-2172-10-26

**Published:** 2009-05-08

**Authors:** Jeffrey G Shannon, Diane C Cockrell, Kazue Takahashi, Gregory L Stahl, Robert A Heinzen

**Affiliations:** 1 *Coxiella *Pathogenesis Section, Laboratory of Intracellular Parasites, Rocky Mountain Laboratories, National Institute of Allergy and Infectious Diseases, National Institutes of Health, Hamilton, Montana, USA; 2 Center for Experimental Therapeutics and Reperfusion Injury, Department of Anesthesiology, Perioperative and Pain Medicine, Brigham and Women's Hospital, Harvard Medical School, Boston, Massachusetts, USA; 3 Laboratory of Developmental Immunology, Massachusetts General Hospital for Children, Harvard Medical School, Boston, Massachusetts, USA

## Abstract

**Background:**

The obligate intracellular bacterial pathogen *Coxiella burnetii *causes the zoonosis Q fever. The intracellular niche of *C. burnetii *has led to the assumption that cell-mediated immunity is the most important immune component for protection against this pathogen. However, passive immunization with immune serum can protect naïve animals from challenge with virulent *C. burnetii*, indicating a role for antibody (Ab) in protection. The mechanism of this Ab-mediated protection is unknown. Therefore, we conducted a study to determine whether Fc receptors (FcR) or complement contribute to Ab-mediated immunity (AMI) to *C. burnetii*.

**Results:**

Virulent *C. burnetii *infects and replicates within human dendritic cells (DC) without inducing their maturation or activation. We investigated the effects of Ab opsonized *C. burnetii *on human monocyte-derived and murine bone marrow-derived DC. Infection of DC with Ab-opsonized *C. burnetii *resulted in increased expression of maturation markers and inflammatory cytokine production. Bacteria that had been incubated with naïve serum had minimal effect on DC, similar to virulent *C. burnetii *alone. The effect of Ab opsonized *C. burnetii *on DC was FcR dependent as evidenced by a reduced response of DC from FcR knockout (FcR k/o) compared to C57Bl/6 (B6) mice. To address the potential role of FcR in Ab-mediated protection in vivo, we compared the response of passively immunized FcR k/o mice to the B6 controls. Interestingly, we found that FcR are not essential for AMI to *C. burnetii *in vivo. We subsequently examined the role of complement in AMI by passively immunizing and challenging several different strains of complement-deficient mice and found that AMI to *C. burnetii *is also complement-independent.

**Conclusion:**

Despite our data showing FcR-dependent stimulation of DC in vitro, Ab-mediated immunity to *C. burnetii *in vivo is FcR-independent. We also found that passive immunity to this pathogen is independent of complement.

## Background

*Coxiella burnetii *is an obligate intracellular bacterium that causes the zoonotic disease Q fever. Acute Q fever typically manifests as an incapacitating, flu-like illness with symptoms including high-grade fever and periorbital headache [1]. *C. burnetii *can persist in its host in a latent state and may reactivate to cause chronic Q fever months or years after initial exposure [2]. Historically, several different Q fever vaccines have been developed, the most successful of which has been an Australian vaccine, Q-vax, that consists of formalin inactivated *C. burnetii *[3]. One dose of Q-vax provides long-lived protective immunity [4]. However, this vaccine can cause severe side effects in recipients with previous exposure to *C. burnetii *necessitating skin testing to determine the immune status of potential vaccinees prior to vaccination. Thus, there is a clear need for a safe, effective subunit vaccine that eliminates the need for pre-testing.

Despite the effectiveness of Q-vax, little is known about the immune mechanisms responsible for the protective immunity elicited by this vaccine. Due to the intracellular niche of *C. burnetii*, it has long been thought that cell-mediated immunity (CMI) must be required for protection against this pathogen. In support of this idea, Andoh *et al*. [5] recently found T cells and interferon-γ are essential for resolution of a primary *C. burnetii *infection. While CMI plays an important role in immunity to *C. burnetii*, passive immunization studies, where serum from vaccinated animals is transferred into naïve animals, clearly demonstrate that Ab alone is capable of providing complete protection in an immunocompetent animal [6-10]. The development of potential subunit vaccine candidates would benefit from a deeper understanding of the precise mechanisms responsible for AMI to *C. burnetii*.

Antibody can provide protection against intracellular pathogens via a number of different mechanisms. These include direct bactericidal activity, complement activation, opsonization, cellular activation via Fc or complement receptors, and Ab-dependent cellular cytotoxicity [11]. Here, we have examined the potential contributions of FcR and complement in AMI to *C. burnetii*.

## Results

### Antibody opsonization does not affect *C. burnetii *viability or replication within phagocytic cells

**Figure 1:**
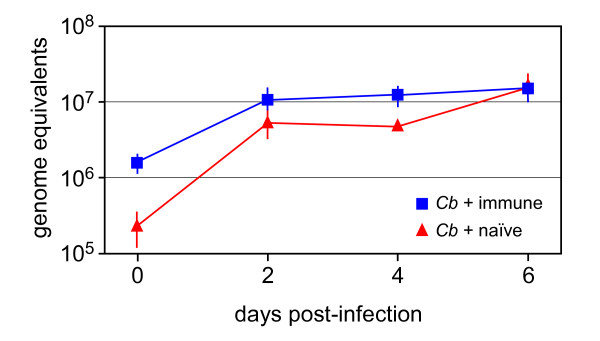
**Growth of Ab-opsonized *C. burnetii *in human macrophages**. *C. burnetii (Cb) *were incubated with naïve or immune human sera, washed and added to cultures of human monocyte-derived macrophages. Bacterial genome equivalents in the culture were quantified at 0, 2, 4 and 6 days post-infection by qPCR. The results shown are from one experiment done in triplicate and representative of three independent experiments.

Ab can mediate protective immunity against bacterial pathogens through direct bactericidal effects or by activation of the complement cascade leading to membrane attack complex deposition on the bacterial surface [12,13]. There are published data showing that neither *C. burnetii*-specific antibodies [14-16] nor complement [17] are directly bactericidal towards virulent *C. burnetii*. To confirm this, we determined whether Ab opsonization affects replication in human macrophages (MΦ), an in vitro model of *C. burnetii *infection [18]. We infected human monocyte-derived MΦ with virulent phase I *C. burnetii *that had been incubated with naïve human serum or immune serum from a chronic Q fever patient containing high titers of anti-*C. burnetii *antibodies and measured bacterial replication over 6 days by quantitative PCR. While Ab opsonized bacteria were taken up more efficiently by MΦ, there was little difference in bacterial yield between Ab-opsonized and non-opsonized *C. burnetii *with ~10^7 ^genome equivalents observed in each case 6 days post-infection (Fig. 1). We also tested the viability of *C. burnetii *after incubation with specific Ab, complement or both and observed no affect (data not shown). Taken together, these data demonstrate that the traditional bactericidal and opsonic mechanisms associated with Ab are not likely to play significant roles in AMI to *C. burnetii*.

### Antibody-opsonized *C. burnetii *stimulate human DC maturation and inflammatory cytokine production

**Figure 2:**
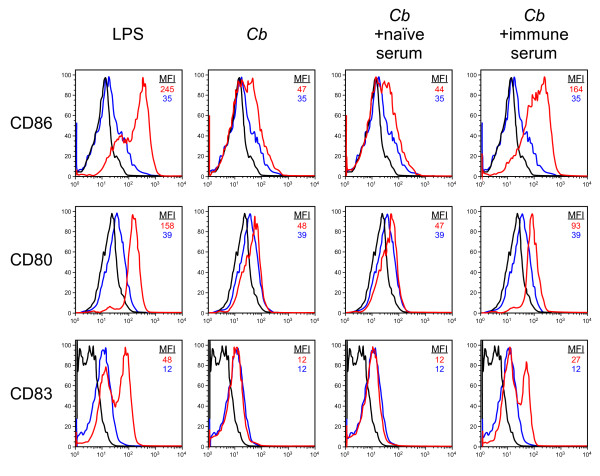
**Stimulation of human DC maturation by Ab-opsonized *C. burnetii***. Human monocyte-derived DC were infected for 24 h with *C. burnetii *(*Cb*), *C. burnetii *that had been incubated with naïve human serum (*Cb *+ naïve), or *C. burnetii *that had been incubated with immune human serum (*Cb *+ immune). Treatment of DC with *E. coli *LPS served as a positive control for maturation. DC maturation was indicated by increased expression of the maturation markers CD86, CD80 and CD83 as determined by flow cytometry. Blue histograms represent untreated or uninfected control DC. Red histograms represent LPS treated or infected DC. Black histograms represent isotype controls. The mean fluorescence intensity (MFI) of untreated or uninfected DC and LPS-treated or infected DC are shown. The results shown are from one experiment and representative of three independent experiments.

**Figure 3:**
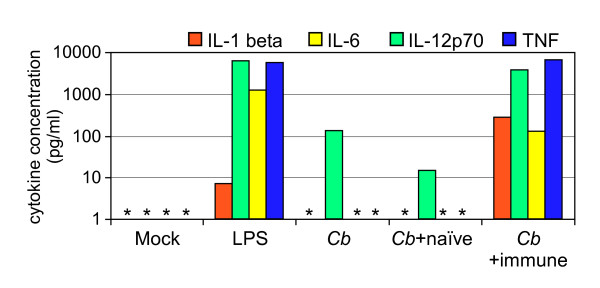
**Stimulation of human DC cytokine production by Ab-opsonized *C. burnetii***. Human monocyte-derived DC were infected for 24 h with *C. burnetii *(*Cb*), *C. burnetii *that had been incubated with naïve human serum (*Cb *+ naïve), or *C. burnetii *that had been incubated with immune human serum (*Cb *+ immune). Treatment of DC with *E. coli *LPS served as a positive control for stimulation. The production of IL-1β, IL-6, IL12p70 and tumor necrosis factor (TNF) by human DC was determined by BioPlex multiplex cytokine assay. Levels below the limits of detection of the assay are indicated with an asterisk. The results shown are from one experiment and representative of three independent experiments.

Virulent phase I strains of *C. burnetii *that express full-length LPS molecules infect and replicate within DC without inducing DC maturation or inflammatory cytokine production [19]. We hypothesized that Ab opsonization of the bacteria would increase their ability to stimulate DC. To test this, we infected human monocyte-derived DC with phase I *C. burnetii *that had been incubated with either naïve or immune human serum. Consistent with our previous findings, infection with untreated control *C. burnetii *did not result in DC maturation (Fig. 2) or inflammatory cytokine production (Fig. 3). Likewise, *C. burnetii *that had been incubated with naïve human serum failed to stimulate the DC. However, infection with *C. burnetii *that had been incubated with immune serum resulted in DC maturation and inflammatory cytokine production similar to that observed after treatment of cells with *E. coli *LPS (Fig. 2 and Fig. 3). Bacteria opsonized with heat-inactivated immune serum stimulated equivalent DC maturation and activation, indicating that complement was not involved in this phenomenon (data not shown). Thus, Ab opsonization of virulent *C. burnetii *dramatically increases the response of DC to this pathogen.

### Stimulation of mouse bone marrow-derived DC (BMDC) by Ab-opsonized *C. burnetii *is FcR-dependent

**Figure 4:**
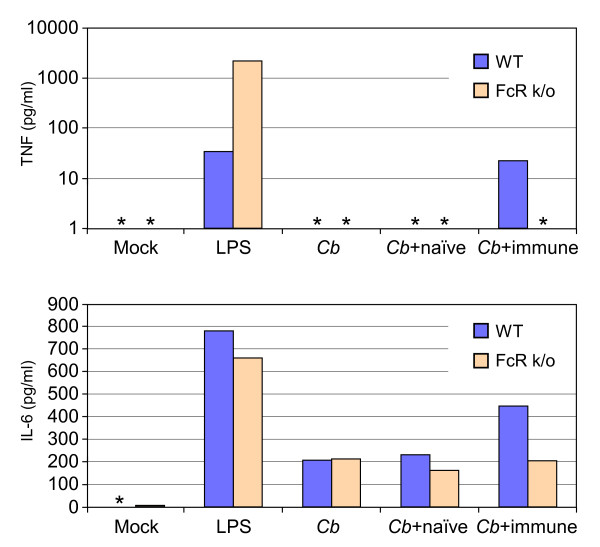
**Response of C57Bl/6 and FcR k/o murine DC to Ab-opsonized *C. burnetii***. Bone marrow-derived dendritic cells were generated from B6 or FcR k/o mice. The cells were mock infected, or infected for 24 h with *C. burnetii *(*Cb*), *C. burnetii *that had been incubated with naïve mouse serum (*Cb *+naive) or *C. burnetii *that had been incubated with mouse immune serum (*Cb *+immune). Treatment of DC with *E. coli *LPS served as a positive control for stimulation. The concentrations of TNF and IL-6 in the DC supernatants were determined by BioPlex multiplex cytokine assay. Levels below the limits of detection of the assay are indicated with an asterisk. The results shown are from one experiment and are representative of three independent experiments.

DC express multiple FcR on their surface and Ab-antigen complexes can stimulate DC through their interactions with a subset of these receptors [20]. We therefore examined the role of these receptors in stimulation of DC by Ab-opsonized *C. burnetii *in vitro. FcR can be divided into activating FcR and inhibitory FcR. The activating FcR associate with a common γ-chain containing an immunoreceptor tyrosine-based activation motif [20]. To determine the role of activating FcR in the response of DC to *C. burnetii *opsonized with mouse immune serum, we infected BMDC from C57Bl/6 (WT) or a common gamma chain knockout mouse strain (FcR k/o), and measured inflammatory cytokine production. Similar to what we observed with human DC, WT mouse BMDC secreted increased amounts of the inflammatory cytokines TNF and IL-6 in response to Ab-opsonized *C. burnetii *(Fig. 4). However, BMDC derived from FcR k/o mice showed greatly reduced cytokine production in response to Ab-opsonized bacteria. These data suggest that the effects of Ab-opsonized *C. burnetii *on DC in vitro are FcR-dependent.

### AMI to *C. burnetii *in vivo is FcR-independent

**Figure 5:**
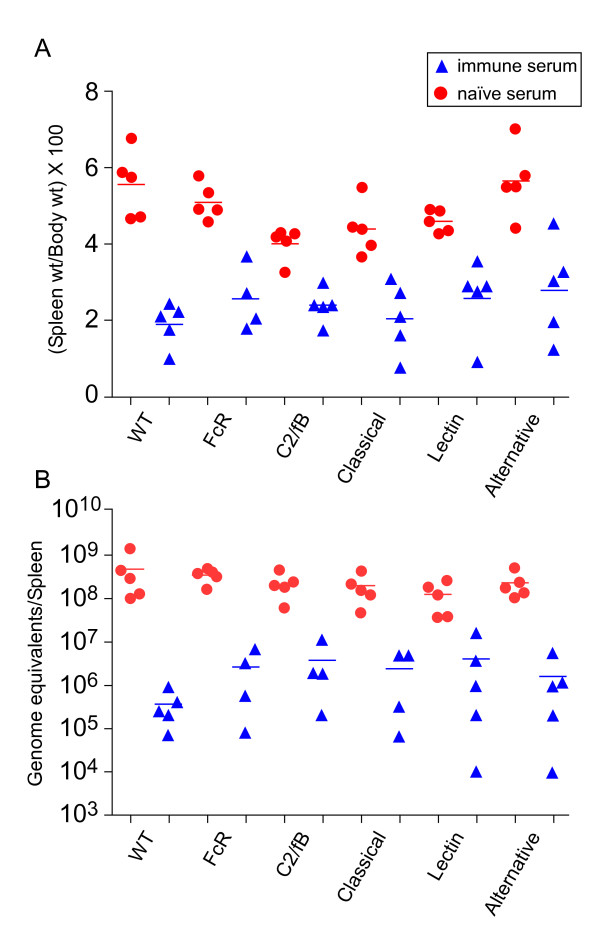
**Passive immunization of FcR and complement deficient mice**. C57Bl/6, FcR k/o, C2/fB double k/o (completely complement deficient), Classical (possessing only the classical complement pathway), Lectin (possessing only the lectin complement pathway), and Alternative (possessing only the alternative complement pathway) mice were injected i.p. with 300 μl of either naïve or immune mouse serum 1 day prior to i.p. challenge with 10^5 ^*C. burnetii*. Severity of infection was determined at 2 weeks post-challenge by assessing spleen weights (A) and bacterial genome equivalents per spleen (B).

Because of the dramatic, FcR-dependent effect of Ab-opsonized *C. burnetii *on DC in vitro, we wanted to determine the role of activating FcR in AMI to *C. burnetii *in vivo. We used a mouse model of Q fever where mice are challenged with 10^5 ^*C. burnetii *bacteria by the intraperitoneal route [21]. Severity of infection was determined by measuring increases in spleen weight and numbers of bacterial genome equivalents in the spleen at 2 weeks post-infection. We tested the ability of Ab to protect WT or FcR k/o mice by passively immunizing mice with mouse immune or naïve serum 1 day prior to challenge. Interestingly, WT and FcR k/o mice were equally protected by passive administration of immune serum. Both strains showed a similar difference in spleen size and bacterial genomes per spleen between naïve serum or immune serum-treated mice (Fig. 5A and 5B). These data indicate that FcR-dependent stimulation of DC, or any other FcR-bearing cell type, is not essential for AMI to *C. burnetii*.

### Role of complement in Ab-mediated immunity to *C. burnetii*

The complement system is important for AMI to a variety of pathogens [22]. Ab-opsonized bacteria can activate the complement cascade which can lead to clearance of resultant immune complexes. To examine the role of complement in AMI to *C. burnetii*, we passively immunized and challenged several different complement-deficient mice. We tested mice deficient in all three complement pathways, i.e., classical, alternative and lectin pathways, (C2/factorB double knockout), the alternative and lectin pathways (Classical mouse, MBL/factorD double knockout), the classical and lectin pathways (Alternative mouse, MBL/C1q double knockout), and the classical and alternative pathways (Lectin mouse, C1q/factorD double knockout). Among the immune serum treated animals, we observed no statistical difference in spleen weight or bacterial genome numbers per spleen between any of the complement-deficient mouse strains and the WT mice (Fig. 5A and 5B). We conclude from these data that AMI to *C. burnetii *is complement-independent.

## Discussion

Despite the obligate intracellular niche of *C. burnetii*, passive transfer of Ab to a naïve, immunocompetent animal provides complete protection against challenge with this pathogen. We sought to determine the specific immune mechanisms responsible for Ab-mediated protection against *C. burnetii*. The fact that Ab alone can protect against *C. burnetii *challenge is even more intriguing because this organism replicates within a phagolysosome-like compartment of mononuclear phagocytes. Therefore, the opsonic mechanisms that are traditionally thought to target infectious organisms to phagocytes where they are destroyed in phagolysosomes are not likely to play a role in AMI to *C. burnetii*. In support of this, we show here that Ab opsonization does not directly affect bacterial viability or intracellular replication of *C. burnetii *in human MΦ. Additionally, our passive immunization experiments with FcR k/o mice indicate that traditional opsonic mechanisms, which are typically FcR-dependent, are not essential for AMI to *C. burnetii*.

There are conflicting reports of the ability of Ab opsonized *C. burnetii *to survive in phagocytes. Hinrichs and Jerrells [15] found that immune serum opsonization of *C. burnetii *increased bacterial uptake by guinea pig macrophages, but had no negative effect on the intracellular replication of the organism. Likewise, Kazar et al. [16] and Baca et al. [14] found that macrophages were unable to control the growth of Ab-opsonsized *C. burnetii*. However, a study published by another group showed that immune serum opsonization allowed for increased uptake and destruction of *C. burnetii *by guinea pig MΦ [23]. Our data show that human monocyte-derived macrophages do not control growth of Ab-opsonized *C. burnetii *in vitro. In interpreting these disparate results, it is important to keep in mind that these are in vitro studies that may not accurately reflect what is occurring in vivo. Further studies involving histological analysis of the fate of Ab-opsonized bacteria in vivo are clearly needed.

There have been several reports of protection of naïve animals from *C. burnetii *challenge by passive immunization with immune serum. Interestingly, T cell deficient mice are not protected by passive Ab transfer [9,10]. Thus, whatever the mechanisms of AMI to *C. burnetii *may be, they are dependent of the presence of T cells. DC express FcR, are stimulated by immune complexes, and are potent stimulators of naïve T cells. Therefore, we examined the response of DC to Ab-opsonized *C. burnetii *and found that DC are stimulated by Ab-opsonized *C. burnetii *in FcR-dependent manner. This led us to examine the role of FcR in AMI to *C. burnetii *in vivo. Given the results of our in vitro experiments, we were surprised to find that AMI to *C. burnetii *was totally independent of activating FcR. Thus, the FcR-dependent stimulation of DC, or any other cell type, by Ab-opsonized bacteria is not essential for protection.

The common γ-chain FcR k/o mouse used in our studies lacks expression of functional activating FcR. However, these mice still express the inhibiting FcRIIb that can bind immune complexes. It would be counterintuitive to expect an inhibiting FcR to contribute to protective immunity to a bacterial pathogen. However, there is one study showing that both activating and inhibiting FcR need to be knocked out in order to observe a defect in AMI to *Bordetella bronchiseptica *[24]. Thus, *C. burnetii *may be similar in that both types of FcR need to be knocked out to see a defect in AMI. Interestingly, AMI to *B. bronchiseptica *is also dependent on a functional complement system [25], which is not true for *C. burnetii*. Therefore, the mechanisms involved in AMI to *C. burnetii *appear more complex than what is known about AMI to other pathogens.

There are several possible mechanisms by which FcR-deficient mice could be protected by passive administration of Ab. Immune sera against bacterial pathogens like *Neisseria meningitidis *and *Borrelia burgdorferi *are bactericidal towards the organism [26,27]. For *B. burgdorferi*, Ab in immune serum can be directly bactericidal whereas, in the case of *N. meningitidis*, the bactericidal activity of immune serum is dependent on a combination of Ab and complement. Our own data and those reported previously [14-16] show that *Coxiella*-specific antibodies from immune individuals or animals are not directly bactericidal. Indeed, Ab enhances uptake of the bacteria by cells and therefore promotes bacterial replication in vitro. Therefore, Ab opsonization does not affect the replication of *C. burnetii*. Furthermore, the combination of anti-*Coxiella *Ab and complement does not affect bacterial viability and the complement cascade is not essential for Ab-mediated protection in vivo.

There are several mechanisms by which complement can contribute to AMI. The C1q component of the classical complement pathway can recognize and bind to the Fc portion of Ab leading to activation of the classical complement cascade and deposition of complement on the surface of immune complexes. Mannose-binding lectin, a component of the lectin pathway of complement activation, can also bind to the Fc portion of IgM [28]. The data presented here demonstrate that none of the pathways of complement activation play essential roles in AMI to *C. burnetii*.

It is possible that in the absence of activating FcR, the complement system can compensate and allow for passive protection of FcR-deficient animals and vice versa. Thus, it would be interesting to examine passive immunity in animals deficient in both FcR and complement.

## Conclusion

The goal of this study was to determine the mechanism(s) by which Ab contributes to immunity to *C. burnetii*. Interestingly, despite our in vitro data indicating a potential role for FcR, we were unable to define a mechanism of AMI to *C. burnetii *in vivo. Thus, the role of Ab in protective immunity to this organism may be more complex than what is seen with other pathogens and warrants further investigation.

## Methods

### *C. burnetii *Purification and Antiserum Production

*C. burnetii *Nine Mile phase I (RSA493) was propagated in African green monkey kidney (Vero) fibroblasts and purified by Renografin density gradient centrifugation [18]. Purified bacteria were suspended in K-36 buffer (0.1 M KCl, 0.015 M NaCl, 0.05 M K_2_HPO_4_, 0.05 M KH_2_PO_4_, pH 7.0). Murine anti-*Coxiella *antiserum was prepared by immunizing C57Bl/6 mice i.p. three times at 2 wk intervals with 10^8 ^fixed *C. burnetii. C. burnetii *were fixed with 4% paraformaldehyde for 24 h at 4°C. The bacteria were washed three times with phosphate buffered saline (PBS, Invitrogen, Carlsbad, CA) and resuspended in PBS at the desired concentration. Immune sera were harvested 2 wks after the final immunization and pooled. Serum from unvaccinated mice was harvested as naïve (control) serum. Human serum from a chronic Q fever patient was generously provided by Ted Hackstadt, Rocky Mountain Laboratories, NIAID, NIH.

### Cell Culture

Human monocyte-derived DC were prepared as previously described [19]. Briefly, human PBMC were isolated from buffy coats by centrifugation through a Ficoll-Paque Plus (Amersham Pharmacia Biotech, Uppsala, Sweden) density gradient. Cells were enriched for monocytes (CD14+ cells) using a RossetteSep Monocyte Enrichment Kit (Stem Cell Technologies, Vancouver, Canada). Monocytes were resuspended at 10^6 ^cells per ml in DC medium (RPMI +Glutamax [Invitrogen], 5% FBS, 15 mM Hepes, 0.1 mM non-essential amino acids, 1 mM sodium pyruvate, 100 U/ml penicillin and 100 μg/ml streptomycin) containing IL-4 (10 ng/ml) and GM-CSF (10 ng/ml) (Peprotech, Rocky Hill, NJ) and cultured for 6 days with replacement of half of the medium and addition of fresh cytokines every other day. Non-adherent DC were harvested by centrifugation and suspended in DC medium without antibiotics. The resulting cells were determined to be >95% CD11c+/CD209+ by flow cytometry. Monocyte-derived macrophages were generated by culturing monocytes in MΦ medium (RPMI, 10% FBS, and 50 ng/ml M-CSF [Peprotech]) for 7 days. Adherent MΦ were harvested by washing with cold PBS and gentle scraping.

The method used to prepare bone marrow derived DC (BMDC) was adapted from methods described by Inaba *et al*. [29]. Briefly, bone marrow cells were isolated from C57Bl/6 or FcR k/o mouse femurs and cultured in DC medium containing 20 ng/ml murine GM-CSF (Peprotech) at 37°C, 5% CO_2_. After 3 days, the culture medium and non-adherent cells were removed and replaced with fresh DC medium containing fresh GM-CSF. The cells were cultured with replacement of half of the medium and addition of fresh cytokines every third day. After 6–8 days of culture, BMDC were harvested as non-adherent cells. These cells were determined to be >80% CD11c+/CD11b+ by flow cytometry.

For *C. burnetii *infection experiments, DC were plated in 24-well plates at a concentration of 5 × 10^5 ^per ml in DC medium. Purified *C. burnetii *were added directly to the culture medium at a multiplicity of infection (MOI) of 100. Unless otherwise indicated, the inoculum was not removed. LPS from *E. coli *serotype O111:B4 (Sigma, St. Louis, MO) was used at 500 ng/ml as a positive control for DC stimulation/maturation. Cells were mock-infected with K-36 buffer. For in vitro experiments in which *C. burnetii *were Ab opsonized, approximately 20 μl of a *C. burnetii *suspension (approximately 10^8 ^bacteria) were mixed with 50 μl of naïve or immune serum (human or mouse) and incubated for 30 min at room temperature. Bacteria were washed twice with PBS (Invitrogen) and resuspended at the desired concentration in PBS.

### Mouse Strains

C57Bl/6 and Fc receptor common gamma chain k/o (Fcer1g) [30] mice on a C57Bl/6 background were obtained from Taconic Farms (Germantown, NY). Breeding colonies of C2/factorB double k/o [31] mice were maintained at Taconic Farms. MBL/C1q double k/o, MBL/factorD double k/o and C1q/factorD double k/o mice were generated by interbreeding MBL k/o [32], C1q k/o [33], and factorD k/o [34] mice and maintained at Taconic Farms. All complement knockout mice were on the C57Bl/6 background (backcrossed at least 10 generations).

### Passive Immunization Experiments

On day -1, mice were injected with 300 μl naïve or immune serum via the intraperitoneal (i.p.) route. On day 0, mice were challenged i.p. with 10^5 ^*C. burnetii*. Mice were sacrificed on day 14, spleens were harvested and blood was collected. Severity of infection was determined by measuring spleen weight and bacterial genomes per spleen. All procedures and animal protocols used in this study were approved by the Biosafety and IACUC committees at Rocky Mountain Laboratories. Work with viable *C. burnetii *was conducted in either BSL-3 or ABSL-3 laboratories.

### Quantitative PCR

*C. burnetii *replication during infection of MΦ was quantified using TaqMan quantitative PCR (QPCR) of genome equivalents as previously described [35]. Briefly, MΦ were incubated with *C. burnetii *(MOI = 100) for 24 h at 37°C, 5% CO_2_. Non-adherent bacteria were removed by washing MΦ with Hank's balanced salt solution and the medium replaced. This point was considered 0 h post-infection. MΦ were harvested by scraping and DNA was isolated with an UltraClean microbial DNA isolation kit (MoBio Laboratories, Carlsbad, CA). For determination of bacterial genome numbers in the spleen, a small portion of spleen tissue (<50 mg) was excised from the spleen, weighed, and DNA extracted using the same DNA isolation kit. The primers/probe set used was designed with PrimerExpress software (Applied Biosystems, Foster City, CA) and is specific for the *C. burnetii dotA *gene. The forward and reverse primers are GCGCAATACGCTCAATCACA and CCATGGCCCCAATTCTCTT, respectively, and the probe sequence is CCGGAGATACCGGCGGTGGG. Purified plasmid DNA containing the *C. burnetii dotA *gene was used as template to generate standard curves ranging form 10^3 ^to 10^8 ^plasmid copies.

### Flow Cytometry

Human DC were harvested by incubation for 5 min in cold 0.1 M EDTA in PBS, followed by gentle scraping and centrifugation at 500 × *g *for 5 min at 4°C. The following monoclonal antibodies were used to characterize DC phenotypes: antiCD83-APC, antiCD86-PE, antiCD209-PerCP-Cy5.5, antiCD80-FITC, and antiCD11c-PE (BD PharMingen, San Diego, CA). Approximately 5 × 10^5 ^DC were stained with antibodies in FACS Wash (PBS, 2% FBS) for 15 min on ice, washed twice with FACS Wash and fixed with 4% paraformaldehyde. Samples were run on a FACS-Canto II flow cytometer (Becton Dickinson, San Jose, CA) and analyzed using FlowJo software (TreeStar, Ashland, OR).

### Cytokine Assay

The concentrations of mouse IL-6 and TNF or human IL-1β, IL-6, IL-12p70 and TNF in culture supernatants were determined by BioPlex X-plex multiplex cytokine assay (BioRad, Hercules, CA) according to the manufacturers instructions.

### Statistics

Statistical analysis was conducted to evaluate the significance between differences. The Student's t-test was used to compare the difference between groups. A *p *value of <0.05 was regarded as statistically significant.

## Authors' contributions

JGS conceived the study, designed and conducted experiments and drafted the manuscript. DCC participated in study design and carried out qPCR assays and animal work. GLS participated in experimental design and animal work. KT generated knock-out mice used in the study. RAH participated in study design and coordination and helped to draft the manuscript. All authors read and approved the final manuscript.
